# Comparative Tests of Infant Foods

**Published:** 1886-10

**Authors:** 


### *COMPARATIVE TESTS OF INFANT FOODS.*

W. H. Rassman, M. D., Attending Physician, North Eastern Dispensary and Late House Surgeon, Maternity Hospital, New York City, under date of Aug. 1, 1886, reports as follows: "Impressed with the importance of the proper feeding of infants, I determined to make as thoroughly as possible a series of comparative clinical tests, both in the North Eastern Dispensary, and in private practice. I obtained eleven varieties of Food, using of each one or more packages, according to the duration of the case. I used as little medicine as possible, and took particular care to have the directions on each package of Food carefully followed. Some Foods I found absolutely worthless if not injurious, containing undigested starch and other elements. Others seemed useful in simple mal-nutrition, but were too laxative and irritating, in their action to be safe in intestinal derangements. The Food, however, which in any and every case fulfilled all requirements was Lactated Food. In malnutrition it was "a complete substitute for mother's milk," acting in a way which charmed the mother, and was highly appreciated by the physician. In the exhaustion consequent on summer diarrhœa and entero-colitis, its effect was wonderful, filling out the emaciated body and checking the disease with little or no medicine. In cholera infantum, however, it achieved its greatest triumph, holding the disease in check in a grand manner, and finally restoring fully the lost weight and strength."

